# Intrinsic Capacities, Functional Ability, Physiological Systems, and Caregiver Support: A Targeted Synthesis of Effective Interventions and International Recommendations for Older Adults

**DOI:** 10.3390/ijerph20054382

**Published:** 2023-03-01

**Authors:** Eleni-Marina Ashikali, Catherine Ludwig, Laura Mastromauro, Samuel Périvier, Aude Tholomier, Irina Ionita, Christophe Graf, Catherine Busnel

**Affiliations:** 1 Geneva Institution for Home Care and Assistance (imad), 1227 Carouge, Switzerland; 2 Geneva School of Health Sciences, HES-SO, University of Applied Sciences and Arts Western Switzerland, 1206 Geneva, Switzerland; 3 Department of Rehabilitation and Geriatrics, Geneva University Hospital, 1226 Geneva, Switzerland; 4 PLATEFORME du Réseau Seniors Genève, 1227 Geneva, Switzerland

**Keywords:** older adults, intrinsic capacities, functional ability, caregiver support, interventions, recommendations

## Abstract

The ageing population calls for interventions that can assist older people to age healthily. This study aimed to provide a targeted synthesis of high-level research and current evidence-based recommendations on effective interventions for maintaining or preventing the decline in intrinsic capacity, functional ability, and physiological systems, or for caregiver support. Nestled within the healthy ageing framework by the World Health Organization, available evidence was selected in a targeted manner, with the purpose of providing a synthesis that would allow the application of this knowledge in real life. As such, the outcome variables were examined through an Evidence and Gap Map of interventions for functional ability and through guidelines from leading institutions. Systematic reviews, meta-analyses, and guidelines on community-dwelling older adults with or without minor health limitations were considered. Thirty-eight documents were included and over fifty interventions identified. Physical activity interventions were consistently effective across several domains. Recommendations point to screening, whilst highlighting the importance of behavioural factors in the endeavour to age healthily. There is a wide range of activities which are likely to foster healthy ageing. To encourage their uptake, it is important for communities to offer suitable promotion and support, and to make these accessible to the public.

## 1. Introduction

The world population is growing older, with people aged 65 and over constituting the most rapidly growing age group [1]. It is estimated that by 2050, twenty-five per cent of the population in Europe and North America could fall within this age group, while the number of individuals aged 80 and above is expected to triple during that same time period [1]. Meanwhile, people report wanting to live at home as long as possible [2,3], and policies on ageing in place have been developed across different countries [4]. While the increase in life expectancy and the opportunities of ageing in place are recognized as positive consequences of important scientific, technological, and medical advancements, they also bring to light economic and social challenges. This highlights the need for effective ways in which to maintain health and independence amongst older adults [5].

In the context of the ageing population and the consequent need for structural and social adaptations [6], the World Health Organization (WHO) developed the Integrated Care for Older People (ICOPE) approach. This approach calls for unified healthcare at the micro-, meso-, and macro-levels [7], placing older adults’ needs at the centre of care by strongly emphasizing case management, and by taking into account their unique situation, their needs and preferences. As such, ICOPE fully complies with the WHO statement that “health is defined, not negatively or narrowly as the absence of disease or infirmity, but positively and broadly as a state of complete physical, mental and social wellbeing “ the enjoyment of which should be part of the rightful heritage of every human being without distinction of race, religion, political belief, economic or social condition” [8] (p. 16). The ICOPE approach is part of a wider framework of healthy ageing, in which the concept of intrinsic capacity, the totality of an individual’s mental and physical reserves, plays a key role. Within this framework, healthy ageing was defined as “the process of developing and maintaining functional ability that enables well-being in older age” [9] (p. 28). Functional ability refers to the attributes which help individuals achieve meaningful living, that is, building and maintaining relationships, learning, growing and making decisions, and being mobile. It is comprised of intrinsic capacity, the environment, and individuals’ interactions with these [10]. In this sense therefore, an individual can continue to experience well-being at an older age, despite declines in physical and mental capacities. With its focus on individual needs and resources and adoption of a person-centred-approach [11], this framework puts forward a positive representation of ageing, one which considers individuals as having capacities that can be optimised. As such, it moves away from the more traditional disease-centred models of health [12,13,14].

In determining the components of intrinsic capacity, the WHO selected domains which represented “clinically important declines in physical and mental capacities, and [were] strong [independent] predictors of mortality and care dependency in older age” [15] (pp. vii-viii). The identified capacity domains are sensory, locomotor, cognitive, vitality, and psychological. Sensory capacities include vision and hearing, locomotor capacity is the ability to move one’s body, and cognitive capacity is the ability to perform mental functions (e.g., memory and language). Vitality includes energy and equilibrium, with nutrition being a key factor for its maintenance, whereas psychological capacity refers to emotional and affective functions, with emphasis placed on depression.

The intrinsic capacity domains are inter-related, suggesting that their building and maintenance throughout the life course may render individuals more resilient and able to prevent disease more easily [16,17,18]. Similarly, declines in one domain may negatively affect others, for example, unaddressed hearing loss has an impact on communication and on cognitive abilities [19,20], which could lead to social isolation and depression [21]. The natural decline in these capacities with age results in a proportion of older adults entering states of more or less pronounced frailty and living with related impairments, such as hearing or mobility loss, cognitive deficits, or malnutrition. In order to allow older persons to maintain their independence, it is crucial to identify effective interventions aimed at preventing further decline within these domains, as well as interventions aiming for the maintenance of the integrity of these capacities before they become compromised [14,22]. Linked to the well-being of older populations are informal caregivers, who play a critical role in care assistance, and one which often comes with burdens which impact on their overall health [23]. For these reasons, adequate social support systems for caregivers are essential, not only from the caregivers’ perspective, but also for the care receiver [15].

A wealth of research reporting on interventions for healthy ageing has emerged over the past few years, whether addressing the construct as a whole [24,25,26] or focusing on specific aspects of it [27]. At the same time, numerous evidence-based recommendations for healthy ageing have also been put forward by scientific societies and government institutions. In order for this rich evidence to be of practical and effective use to all stakeholders (e.g., researchers, older people, healthcare professionals, and communities), it must be mapped in a manner that allows them to identify “which interventions are supported by evidence, which are not, and which need more research” [28]. In response to this need, the WHO is developing Evidence and Gap Maps (EGM), which are interactive online tools grouping “existing evidence on a given topic” [28]. These tools can, therefore, act as reliable information resources for further research and analysis, as well as for informing current practices and policies.

The aim of the present paper was to provide a targeted synthesis of interventions and recommendations for older adults, using the WHO framework of healthy ageing. More specifically, it focuses on (a) intrinsic capacities (sensory, locomotor, cognitive, vitality, and psychological); (b) functional ability, including the performance of Activities of Daily Living (ADL), Instrumental Activities of Daily Living (IADL), and social relationships; (c) physiological system health; and (d) caregiver support. The choice to expand on the domain of psychological capacity was made such that the symptomatology of anxiety, as well as of depression, was taken into consideration, whereas the domain of vitality placed focus on nutrition. The addition of physiological systems was made to examine health behaviours and primary prevention measures that are effective in delaying the onset of the most common chronic diseases, thus contributing to the preservation and maintenance of intrinsic capacities and functional ability. The focus within this domain was placed on the most important factors in the decline in disability-adjusted life expectancy, notably cardiovascular diseases, cancers, respiratory and musculoskeletal pathologies [29]. To our knowledge, this is the first study to address the full spectrum of the WHO healthy ageing framework, including an expansion of certain domains, through (1) the identification of interventions and determination of their effectiveness, (2) a synthesis of evidence-based recommendations, and (3) from the different points-of-view of health promotion, primary and secondary prevention.

## 2. Materials and Methods

Based on the WHO framework of healthy ageing, this study sought to identify effective interventions and current evidence-based recommendations for older adults, with the wider aim to integrate these findings into a reference guide for use by professionals and individuals. The goal was to adopt a targeted search strategy that would allow for sufficient insight into the scientific consensus on the types of interventions that are considered as effective for older adults.

As such, an EGM of interventions to improve functional ability among adults aged 60 and over was selected as a core document [30]. This EGM is the first of a WHO “multi-year project to summarize, disseminate, and increase uptake of evidence towards deeper integration in policies and practices” [28]. The EGM used a thorough and “comprehensive search that included 11 databases” [31], such as MEDLINE, EMBASE, CINAHL, Cochrane Database of Systematic Reviews, and PsychINFO, as well as a targeted search for grey literature. For the purposes of the present study, systematic reviews and meta-analyses were extracted and screened for the title/abstract by one author (EA), and a second author (LM) screened a random subset of excluded articles (N = 25, ~20%) for double review to confirm exclusion. Two authors (EA and LM) individually read the full text of the remaining articles, eliminating those that did not fit the inclusion criteria (see below). Discrepancies on these decisions were resolved through discussion.

**Figure 1:**
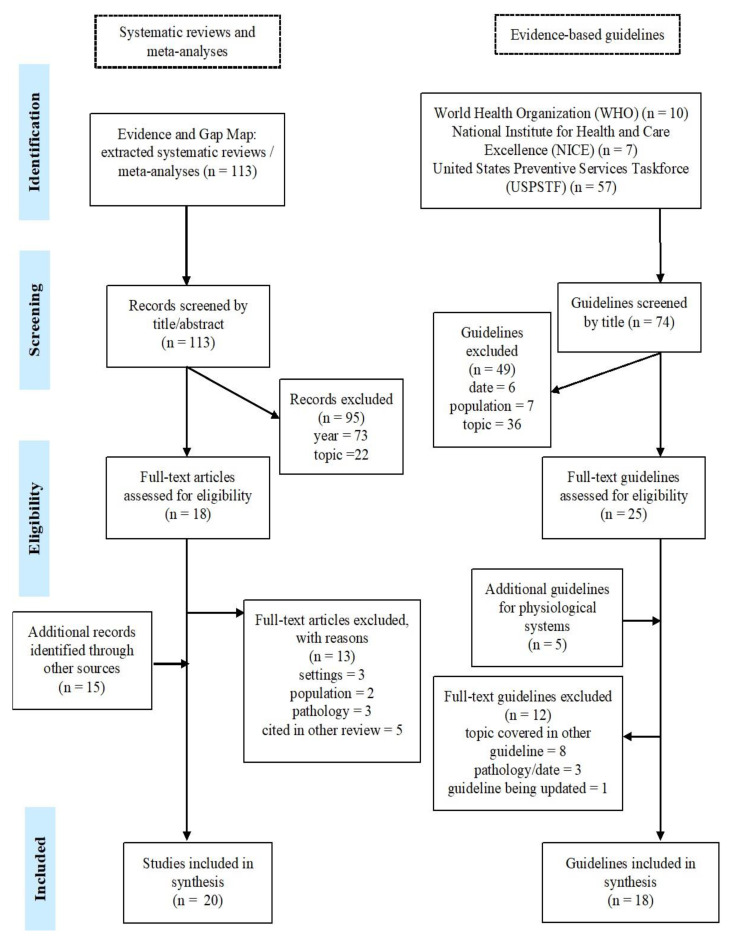
Search strategy and selection process.

To identify evidence-based guidelines, the search terms “older”, “older people”, and “senior” were used in the guideline section of the sites of three institutions: the WHO, the United States Preventive Services Task Force (USPSTF), and the National Institute of Health and Care Excellence (NICE). These institutions were selected due to their extensive research in the field and their status as key institutions in setting healthcare policies and standards. Moreover, the different focus of each institution also played a role in their selection, with the WHO being responsible for setting worldwide healthcare frameworks and guidelines, NICE focusing on healthcare standards, and USPSTF representing a more medical perspective in terms of guidelines. Additional recommendations for physiological system health were identified through specific and specialised associations, such as the European Society of Cardiology. To the extent possible, evidence reports or the published scientific articles were located for data extraction. Figure 1 illustrates the full search strategy and selection process of included research.

Inclusion criteria pertained to the population characteristics and study designs. The target population was community-dwelling (non-institutionalised) people, aged 50 years and over, who were healthy or who presented with limitations in the domain investigated. Studies that included a mix of ages and residential statuses were considered only if the majority of participants were above 50 years old and residing within the community. Included documents were systematic reviews (SRs), meta-analyses (MAs), and evidence-based guidelines published between January 2015 and June 2022 in English, French, Italian, Spanish, or Greek. The primary outcomes of interest were: (a) Intrinsic capacity primary outcomes: vision, audition, locomotor, cognition, nutrition, and psychological (depression and anxiety); (b) Functional ability primary outcomes: ADL/IADL performance and social relationships, through interventions for social participation and a reduction in social isolation and loneliness; (c) Physiological system health: cardiovascular, respiratory, osteoarticular, genitourinary, digestive, and genital systems, and (d) caregiver support. Other study types (e.g., randomised controlled trials and books) were excluded. Populations with chronic illnesses falling within tertiary prevention or in palliative care were excluded. Research on participants living in settings other than the home (e.g., hospitals, nursing homes, and other institutions) was also excluded.

Data from included documents were reviewed and extracted into Excel by one author (EA). Clusters of data extracted included study details (first author, publication year, aims, study design, participants, settings, and outcome variables), search details (year range and number and type of studies included), and results (main findings and conclusions). The documents were examined on an individual basis and categorized according to the main outcomes. They were discussed narratively in terms of the effectiveness of the identified interventions among individuals without limitations (health promotion/primary prevention), then among those presenting with some limitations (secondary prevention).

A specific procedure was followed in order to determine the effectiveness of interventions. First, statistically significant findings from an MA were considered as the strongest indicators of intervention effectiveness, the clear reporting of which required little further examination of the article (see Supplementary Tables S1–S9 for more detailed information on MA findings). Second, in the absence of an MA, if the discussion and conclusion of the SR gave clear indications on the interventions (description and effectiveness), these were reported as such in the present study. Third, if information on intervention effectiveness was unclear from the MA or in the discussion/conclusion of the SR, the results section was examined thoroughly. If an intervention was rated differently in different reviews, the rating in the present paper followed either the strongest evidence (i.e., MA vs. SR), or followed the direction of findings reported in most papers within systematic reviews. Due to the secondary interest of this review in identifying and linking effective interventions to existing real-life activities, in cases where the intervention was not clearly defined and described, further information on the content of interventions was also sought.

## 3. Results

### 3.1. Document Selection

One hundred and thirteen systematic reviews were extracted from the EGM [30]. Out of a total of 33 articles assessed in full text for inclusion eligibility, 20 were included and 13 excluded [32,33,34,35,36,37,38,39,40,41,42,43,44]. Searching the websites of WHO, USPSTF, and NICE led to the identification of 74 evidence-based guidelines, 25 of which were assessed for eligibility at full-text , along with five additional guidelines pertaining to physiological systems. Of these, 18 were included. In total, therefore, 38 documents were included in the present study. Of the articles and guidelines selected for inclusion, four pertained to sensory capacities [45,46,47,48], four to locomotor capacity [49,50,51,52], four to cognitive capacity [53,54,55,56], four to vitality [57,58,59,60], and three to psychological capacities [61,62,63]. In terms of functional ability, three articles covered ADL/IADL performance [64,65,66], and four social relationships [67,68,69,70]. Six documents were related to physiological systems [71,72,73,74,75,76], and two articles were related to caregiver support [77,78]. Finally, two reviews covered a range of the above domains [79,80], one guideline was relevant to both locomotor capacity and physiological systems [81], and another to psychological capacity and social relationships [82].

### 3.2. Document Characteristics

#### 3.2.1. Study Design

An overview of the included documents and relevant characteristics for each outcome of interest is presented in Supplementary Tables S1–S9. Evidence profiles or published articles that were used to create recommendations were found for 13 guidelines and these are reported in the following sections in conjunction with the other research selected for the present study [45,46,49,50,53,57,61,76,77,83,84,85,86]. In total, 14 systematic reviews [52,59,60,65,66,67,68,69,71,78,79,80] and 19 meta-analyses [51,54,55,56,58,62,63,64,85,86], nine of which used a Grading of Recommendations Assessment, Development, and Evaluation (GRADE) [45,46,49,50,53,57,61,76,77] approach, reported on over 600 studies. All reviews included empirical studies, with the majority being randomised controlled trials (N = 372), followed by systematic reviews (N = 54). The remainder were quasi-experimental, cohort, non-randomised and clinical trials, longitudinal, qualitative, and cross-sectional studies. One study included three systematic reports by public health organizations [80] and another a systematic review with meta-analysis [55].

#### 3.2.2. Participants and Settings

The total numbers of participants ranged from 553 to 188,470 per review. The age of participants ranged from 50 to 114, with the majority of reviews considering older adults aged 60 and over. Participants were heterogeneous in terms of health status both between and within different reviews. Pre-existing conditions included visual impairment, hearing loss, mild cognitive impairment, osteoporosis, mobility limitations, sarcopenia, depression, and risk factors for various diseases. Individuals considered as independent, healthy, and free of existing conditions were also included in a number of reviews.

As indicated in the Supplementary Tables S4–S9, individual studies that were carried out among people with pathologies falling within the realm of tertiary health care (e.g., dementia or Parkinson’s disease) [51,56,61,67,77] or those living in care facilities were not considered in the present paper [58,65,66,67].

### 3.3. Summary and Classification of Identified Interventions

**Figure 2:**
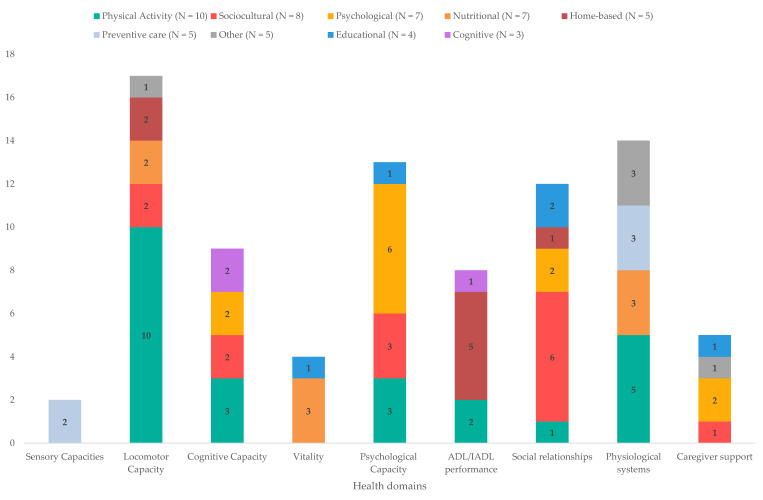
Summary of the types of interventions identified, and the breakdown of the domains they affect.

Fifty-four interventions for health promotion and primary and secondary prevention were identified and classified into the following categories according to the type of intervention: physical activity (e.g., tai chi and yoga; N = 10), sociocultural (e.g., music and art; N = 8), psychological (e.g., psychological therapies and mindfulness; N = 7), nutritional (e.g., nutritional supplements; N = 7), home-based (e.g., home modifications; N = 5), preventive care (e.g., vaccinations and screening; N = 5), other (e.g., respite care; N = 5), educational (e.g., dietary education and technology training; N = 4), and cognitive (e.g., cognitive stimulation; N = 3). Figure 2 provides an overview the types of interventions identified, as well as the domains they have an impact on.

### 3.4. Interventions and Recommendations for Intrinsic Capacity

#### 3.4.1. Sensory Capacities (Vision and Audition)

As sensory capacities can only be improved with medical interventions or external devices (glasses or hearing aids), WHO and USPSTF investigations focused on the detection and screening of decline within these domains (Table 1).

Interventions in the field of eye care are available at the promotion, prevention, treatment, and rehabilitation levels, and are among the most cost-effective and most easily implemented [45]. Updating previous evidence on the benefits of screening for impaired vision within primary care settings, the USPSTF found no direct links between such screening and no screening, usual care, or delayed screening and vision acuity [85]. It concluded that “*the current evidence is insufficient to assess the balance of benefits and harms of screening for impaired visual acuity in older adults*” [47]. Focusing on community case finding and treatment and/or referral of the two most common eye conditions, cataracts and refractive error, some evidence that immediate care for the two conditions leads to improvements in visual acuity was found [45]. Although no evidence supported the effectiveness of community case finding through mass screening, there was an indication that screening and immediate care provision for refractive error and cataract provided benefits to visual function. The WHO recommendation was, therefore, based on screening within primary care, such that “*older people should receive routine screening for visual impairment in the primary care setting, and timely provision of comprehensive eye care*” [45] (p. 17).

Interventions for auditory health at the primary care level include systematic clinical screening at strategic life stages, namely among older adults. Consequently, the USPSFT examined screening for hearing loss in people aged 50 and over, finding no significant differences in hearing-related function between screened and unscreened groups. Moreover, benefits of hearing aids to function were limited to studies which focused on veterans [86]. Therefore, the USPSTF concluded that the “*current evidence is insufficient*” for this domain as well and issued no recommendation [48]. The question investigated by the WHO was focused on how the uptake of hearing aids may be increased in older people with hearing loss. The analysis found that screening for hearing loss did indeed increase the use of hearing aids at the one-year follow-up, whereas the provision of hearing aids had the additional benefits of improved social functioning and communication, as well as decreases in depression. Self-management support interventions showed a significant reduction in self-reported hearing handicap and an increased use of verbal communication. The recommendation that ensued, therefore, was that “*screening followed by provision of hearing aids should be offered to older people for timely identification and management of hearing loss*” [46] (p. 17)

#### 3.4.2. Locomotor Capacity

Locomotor capacity refers to one’s “physical ability to move their body” [2] (p. 170). This capacity, along with the risk of falls, was primarily addressed via physical activity, although other types of interventions have also shown benefits for these domains, such as nutritional or intergenerational interventions (Table 2).

*Interventions for individuals without specific locomotor limitations.* Among older adults not presenting with locomotor limitations, interventions with a social element provided effective opportunities to be physically active. Intergenerational interventions are based on structured programs of a relatively long duration, generally aiming to bring older adults and children together through activities in which the adults can offer their experience. These were considered effective for the maintenance of locomotor capacity through their impact on physical strength and the reduction in sedentary behaviour [80].

A wide range of different types of physical activity have been investigated, including water-based physical exercise, mind–body and multimodal exercises, dance, exercise via video games, home visits, and interventions aiming to reduce sedentary behaviour.

Multimodal exercise, using a combination of exercises, such as strength, flexibility, balance, gait, and endurance, has been found to be the most effective intervention for locomotor capacity. This type of exercise program has shown benefits across a range of outcomes, including physical performance, muscle function and muscle mass, gait, balance, agility, and flexibility [80,81]. Research suggested that the effectiveness of such exercise programs might be affected by the delivery mode, finding evidence that supervised exercise is superior to unsupervised exercise [51]. The authors concluded that among healthy older adults, supervised physical training, by physiotherapists or trained instructors, has greater benefits for balance and muscle strength than unsupervised training. Moreover, such benefits could also be seen when small doses of supervision are incorporated into unsupervised training interventions. Their recommendation was that supervised sessions be included in two out of three training sessions per week [51].

Aquatic physical exercise, including resistance training, muscle activation exercises, flexibility and mobility exercises, was found to be particularly beneficial for physical functioning among older adults without limitations, improving balance, flexibility, muscular strength and mass, agility, and walking capacity, whilst also reducing the fear of falling in sedentary older adults [79]. Research has furthermore suggested that exercising in water is at least as efficient as land-based exercise, with some outcomes, such as postural stability, indicating a higher effectiveness for water-based exercise [80]. These types of interventions are most impactful when individuals engage in them several times a week over the course of several months [80] and when they contain high-intensity exercises [79].

Promising effects have also been found in interventions including yoga, tai chi, dance, as well as sociocultural activities, such as music and singing. Yoga interventions were beneficial for balance and physical mobility, whereas tai chi showed benefits to muscular strength, joint stiffness, physical mobility, and the risk of falls [50,80]. The effectiveness of these types of physical exercise is augmented in intensive, structured programs delivered by a professional trained and certified in the discipline. Similarly, promising effects were found in interventions using video games (exergaming) showing improvements in balance, physical performance, and overall physical activity uptake, with their motivational aspect being considered as an additional potential advantage [80]. Dance interventions showed promise in reducing the fear of falling, while music and singing were promising for the reduction of falls [80].

Programs aiming to reduce sedentary behaviour through regular interruptions of time spent sitting have shown promising effects for physical function and activity level, risk of disability, muscle loss, and cardiometabolic outcomes [80]. Simple strategies can be used for the breaking of sedentary bouts, such as standing up and taking a few steps during TV commercial breaks, standing or moving when speaking on the phone, or using the kitchen counter for reading the morning paper. All of these can be proposed to older people as an easy means of reducing sedentary time during their daily activities, and of enforcing WHO recommendations stating that “*older adults should limit the amount of time spent being sedentary, [and that] replacing sedentary time with physical activity of any intensity…provides health benefits*” [81] (p. 46).

*Interventions for individuals with locomotor limitations and at an elevated risk of falling.* Nutritional, home-based, and physical activity interventions showed positive effects on locomotor capacity among people with limitations.

Mareschal and colleagues sought to systematically document the role of nutritional interventions in the prevention of functional decline among community-dwelling older adults [52]. Their findings showed that nutritional supplements and the combination of nutritional interventions with physical exercise were beneficial for muscle mass, strength, and physical performance among frail and sarcopenic individuals. The latter combination of interventions brought additional benefits in preventing sarcopenia among healthy individuals. Finally, a combination of over two types of interventions (multicomponent interventions) was considered the most beneficial for frail older adults, showing improvements in handgrip strength and a range of other physical performance tests.

Home-based interventions were found to be important for people with locomotor limitations. Home safety interventions, involving the modification of the older person’s environment, led to a significant decrease in falls, particularly when the intervention included an occupational therapist. The WHO issued a recommendation to this effect, such that “*following a specialist’s assessment, home modifications to remove environmental hazards that could cause falls should be recommended*” [50] (p. 24). Occupational therapy (OT) was further highlighted in research showing that home-based multidisciplinary interventions which included OT were effective for gait and balance abilities, as well as for reducing the rate of falls and fear of falling [50,64,80]. These types of interventions were in unimodal or multifactorial formats, the latter including geriatric assessment and active intervention or referral/provision of information. The WHO concluded that “*multifactorial interventions integrating assessment with individually tailored interventions can be recommended to reduce the risk and incidence of falls among older people*” [50] (p. 24).

In terms of physical activity interventions, tai chi was shown to be effective for improving balance, whereas progressive resistance training (PRT) showed positive effects for muscle mass and strength, body weight [81], and performance on physical strength tests [49]. Multimodal exercise touched a wider array of physical domains, significantly improving balance, muscle strength, physical functioning, functional lower extremity strength, and ADL performance [49]. This type of exercise was also effective in reducing the rate of falls, fall-related injuries, and the fear of falling [50,81]. The recommendation put forward by the WHO for individuals with limitations in physical function and at an elevated risk of falling, therefore, was that “*multimodal exercise, including PRT and other exercise components (balance, flexibility and aerobic training), should be recommended for older people with declining physical capacity, measured by gait speed, grip strength and other physical performance measures*” [49] (p. 22). Finally, the rate of falls was also reduced with the removal of psychotropic medications, prompting the recommendation of “medication review and withdrawal (of unnecessary or harmful medication)” [50] (p. 24).

#### 3.4.3. Cognitive Capacity

“Cognitive capacity refers to a person’s capacity to perform a range of mental functions, including…memory, language,…decision-making, [and] abstract thinking” [31] (p. 170). Effective interventions for cognitive capacity in older adults include physical exercise, music, intergenerational and psychosocial activities, cognitive training, and cognitive stimulation (Table 3).

*Interventions for individuals without cognitive limitations.* Physical exercise programs [56,80], including resistance training, yoga, and aquatic exercise [56,79], were shown to be effective for cognitive function in terms of visuospatial abilities, verbal short-term memory, and working memory [56], whereas dance has shown promising effects for global cognition and memory [55]. In their comparative analysis of different types of interventions, Vaportzis and colleagues [56] concluded that physical activity interventions were more effective for cognition than cognitive activity interventions (e.g., computer training and reading), and that visuospatial abilities may be the area of cognition most susceptible to improvements from physical exercise.

Mind-stimulating activities, such as those in intergenerational programs, cognitive training, or music were rated as effective for the maintenance of cognitive capacities. Cognitive training provided benefits in the skills targeted, with interventions on reasoning and processing speed having a beneficial effect at the 10-year follow-up [80]. Finally, promising effects were found in meditation and mindfulness-based programs in terms of attention, memory, cognitive flexibility, verbal fluency, and executive function [80].

*Interventions for individuals with cognitive limitations.* Interventions involving music were investigated in individuals without cognitive limitations, as well as those with mild cognitive impairment (MCI). Instrument playing was effective for cognitive processing speed, with additional tasks within the intervention offering benefits for different areas of cognition. Immediate cognitive engagement (e.g., instrument playing while creating a new rhythm) was beneficial for general cognition and attentional control, whereas sustained cognitive engagement (e.g., instrument playing while reading a score based on memorized information) was beneficial for executive function and processing speed [54]. Among older adults with some form of cognitive impairment, including MCI, cognitive stimulation showed small consistent benefits to overall cognitive function [53]. This was not the case for cognitive training, due to its focus on specific cognitive domains, the benefits of which do not tend to generalize to other functions. Therefore, it would appear that cognitive stimulation provides general benefits to cognitive function, whereas cognitive training benefits are contained within specific domains. Consistent with this, the recommendation for older adults with cognitive impairments was that “cognitive stimulation can be offered to older people with cognitive impairment, with or without a formal diagnosis of dementia” [53] (p. 16).

#### 3.4.4. Vitality (Nutrition)

Vitality is the ability of the human body to maintain a balance of physical and mental functioning “in the face of usual daily exposures…[or in] unusual challenges, such as injury or infection”. Particular emphasis is placed on nutrition as “one of the key factors in maintaining vitality in older age” [31] (p. 170). Behavioural and educational interventions, along with nutritional supplementation, showed beneficial effects on nutritional status and food intake (Table 4). In this domain, only one review [59] focused on individuals without any kind of limitations.

Zhou and colleagues found meal service interventions to be effective in increasing fruit consumption in low fruit consumers [60]. Dietary education interventions, which included components of nutritional advice or counselling, and tailored educational information also showed benefits in increasing fruit and vegetable frequency consumption and intake, as well as in improving general nutritional status. The authors concluded that dietary education and healthier meal services could lead to improved dietary quality. The authors concluded that dietary education and healthier meal services could lead to improved dietary quality. The benefit of educational dietary interventions, whether individual or in a group setting, were also shown to be effective in other reviews in increasing fruit, vegetable [58,59], fibre, micronutrient [58], and protein [59] intake in older adults who were healthy, frail, or at risk of chronic disease. Interventions targeting specific aspects of nutrition, for example those focused on increasing a specific food type versus improving general nutritional status or involved more than one educational session, were found to be the most effective in healthy older adults [59].

In socially vulnerable people, food aid interventions offering free healthy meals and nutritional education showed promise in improving dietary intake [80]. Similarly, among community-dwelling people who were undernourished or at risk of undernourishment, oral nutritional supplementation (ONS) with or without dietary advice was found to be the most effective intervention, bringing weight gain and improvements in handgrip strength. The WHO recommendation was, therefore, that “ONS with dietary advice should be recommended for older people affected by undernutrition” [57] (p. 23).

#### 3.4.5. Psychological Capacity

Psychological capacity, as conceptualised by the WHO, relates primarily to “emotional functions”, that is, “the mental functions related to the feeling and affective components of the process of the mind” [31] (p. 170). Effective interventions for psychological capacity include physical activity, activities with social elements, and psychological therapies (Table 5).

*Interventions for individuals without limitations.* Anxiodepressive symptomatology showed improvements in a range of interventions involving physical activity or social elements. Physical exercise programs were considered effective for symptoms of both anxiety and depression, whereas intergenerational activities were effective for depressive symptoms alone [80]. Among sedentary older adults, aquatic exercise was effective in improving mood in terms of fatigue and tension, as well as anxiety [79]. Promising effects were found in tai chi, music and singing, and mindfulness-based programs, all of which acted on anxiodepressive symptoms, the latter also eliciting positive affect more generally [80,82]. Finally, there are indications that continued education and learning can provide improvements in well-being, notably in increasing positive affect [82].

*Interventions for individuals with subthreshold depression or with clinical diagnosis of depression or anxiety.* The treatment of clinical depression often involves the use of medication, which, among older populations, increases the risk of polypharmacy, hospitalisation, and medication dependency. Therefore, especially for these populations, nonpharmacological interventions are preferable as the first port of call on appearance or diagnosis of mental health disorders, particularly for individuals presenting with mild symptoms [61].

Mindfulness-based stress reduction (MBSR) was shown to be effective among clinically depressed older people not undergoing other treatment [62]. However, active controls were superior to MBSR and no evidence was found for anxiety. Music therapy showed some promising effects for depression when added to standard care, but was not found to be beneficial on its own, suggesting that its use should be in conjunction with standard care [63].

Finally, in clinical and subthreshold depression, a range of psychological therapies was effective in reducing symptoms [61]. Behavioural activation (BA) was not only found to be effective in reducing depressive symptoms in clinical depression, but it was also considered as potentially beneficial in cases where antidepressants were ineffective. For subthreshold depression, psychological interventions such as cognitive-behavioural therapy (CBT), problem-solving therapy (PST), life-review therapy, interpersonal counselling, and BA, were effective in reducing the symptoms of depression, as well as reducing the incidence of depressive disorder at two, six, and twelve-month follow-ups. The recommendation put forward by the WHO was, therefore, that “*older adults who are experiencing depressive symptoms can be offered brief, structured psychological interventions…delivered by health care professionals with a good understanding of mental health care for older adults*” [61] (p. 18).

### 3.5. Interventions and Recommendations for Functional Ability

With autonomy and social relationships being key aspects to functional ability, this section covers interventions for the performance of Activities of Daily Living (ADLs) and Instrumental Activities of Daily Living (IADLs), and interventions aimed at building and maintaining social relationships.

#### 3.5.1. Activities of Daily Living and Instrumental Activities of Daily Living

Interventions including an occupational therapist, whether as part of a multidisciplinary team or not, were those considered the most effective for improving the performance of ADLs and IADLs among community-dwelling people with a range of difficulties (Table 6).

Multidisciplinary home- and community-based interventions containing occupational therapy (OT) were effective in the improvement in functioning in ADLs [64]. Physical exercise interventions accompanied by an occupational therapist were moderately beneficial for frail older adults, with task-specific exercises being moderately beneficial for individuals with difficulties in ADL performance [66]. For those facing difficulties with ADL performance, high benefits were noted in home-based interventions, including in-home assessments, recommendations, and home modifications. The authors concluded that physical interventions are most beneficial for frail older people, and that home-based interventions are most beneficial for individuals with difficulties in ADL performance [66].

Tailored multidisciplinary interventions containing OT were also beneficial for IADL performance, with strong evidence being found among cognitively intact individuals and those affected by MCI [65]. For example, functional cognitive interventions focused on memory, attention, and problem solving were found to be superior to cognitive training in terms of improving cognition, and had lasting benefits on IADL performance. Home-based multidisciplinary rehabilitation interventions slowed down the decline in IADL performance, improved mobility, and led to better engagement with activities. Finally, self-management and preventive home-based interventions were beneficial for the enhancement of IADL performance, with the latter also impacting functional disability.

#### 3.5.2. Social Relationships

Interventions focused on social participation or loneliness reduction were in the format of individual, group, or mixed activities, containing elements of education, training, social support, technology, and psychological and occupational therapy [64,65,67,68,69,80,82] (Table 7).

Through the maintenance of social links, social engagement, activity participation, perceived social support, and the reduction in feelings of isolation, intergenerational programs were rated as effective for social health [80]. Physical activity, professionally led group exercise [67], and interventions containing OT [64,65] were also considered effective for social health. Finally, also effective was a range of educational interventions focused on the maintenance and enhancement of social networks [67]. Examples of interventions included computer training, training on caregiver relationships and on social networks, and visual art discussions [67].

Psychological interventions, notably psychoeducation, CBT [70], and mindfulness [68,78], were shown to offer promising effects for the reduction in loneliness, whilst a range of shared activities with a psychosocial component also showed promise. Music, chorale participation, arts and crafts, and a range of support groups were considered as offering promising options for the reduction in loneliness by allowing older people to build friendships and work towards shared goals, whilst also using their creativity and developing meaningful roles [67,68,80,82].

Increased effectiveness was noted when interventions were carried out in public or social spaces, delivered by professionals in the relevant field (e.g., health commissioners and social workers) [69], and were based on shared interests [70]. An educational approach [67,70], elements of social support [68], and the active participation of older people [69,70,82] were also thought to increase effectiveness. Finally, technology, whether in its use (e.g., online support groups) or in training related to it (e.g., lessons on internet use), has been highlighted as a promising additional value in the reduction in social isolation and the fostering of social participation on a digital scale [67,70,82].

In line with the above research, guidelines for the autonomy and mental well-being of older adults recommend multicomponent group activities, including singing programs, creative activities, intergenerational activities, and community-based physical activity, or one-to-one activities including volunteering, befriending services, or training programs on the development and sustenance of friendships [70,82]. The guidelines also recommend that communities consider offering “*activities, training and ongoing support that encourages older people to use information and communication technologies*” [82] (p. 7).

### 3.6. Interventions and Recommendations for Physiological System Health

Recommendations and interventions for physiological system health in terms of primary care addressed behavioural factors, such as smoking and alcohol consumption, and were in the nature of preventive care (i.e., screening and vaccinations) or in the domains of physical activity and nutrition (Table 8).

#### 3.6.1. Behavioural Factors

As a strong determinant of healthy ageing, physical activity and related interventions have been extensively investigated, with research finding an “*inverse dose–response relationship between volume of aerobic physical activity and risk of physical functional limitations*” [81] (p. 55). In general, therefore, the more engagement with physical activity in terms of frequency, duration, and/or volume, the greater the benefits to physical functioning. Recent guidelines from the WHO and the European Society of Cardiology (ESC) [74,81] state that all older adults, including those without mobility issues, should engage in 150–300 min of moderate-intensity physical activity (e.g., brisk walking, household work, water aerobics, dancing, cycling, or pushing a lawnmower) per week. Alternatively, 75–150 min of vigorous-intensity activity (e.g., running, swimming, aerobics, or walking up the stairs) are recommended weekly. The ESC recommends biweekly engagement in resistance exercise to reduce cardiovascular risk, while both institutions recognize the benefits and importance of multimodal exercise. A key message from these guidelines is that adults should strive to limit the amount of time spent being sedentary and that, in terms of health benefits, some form of activity is better than no activity at all.

Contrary to physical activity, smoking is a significant risk factor for a range of health domains, including sensory and cognitive capacities, for cardiovascular and respiratory diseases, and several cancers. The unanimous consensus and strong recommendation, therefore, is smoking cessation [74], with research showing encouraging evidence of postponement of the onset of cardiovascular episodes by at least two years in quitters aged 70 and over [74]. It is also advised that the assessment of consumption and motivation to quit, as well motivational and/or pharmacological approaches to assist with stopping to smoke, be considered [74,80].

Finally, diet and alcohol consumption also play a role across a range of domains. A balanced diet, namely a Mediterranean-type diet, plant-based and rich in fibre, with reduced saturated fats, salt, and red meat, is recommended for cardiovascular health [74], whereas it is advised to restrict alcohol consumption [72] to a maximum of 100 g per week (equivalent to 7 glasses of wine) [74]. Recommendations for interventions to address alcohol consumption include the initiation of discussion, evaluation, and the use of motivational approaches to help with a reduction in or cessation of consumption [73].

#### 3.6.2. Cardiovascular System

Multimodal exercise [80,81] and dance were considered as effective for aerobic activity (Vo2 max), whereas tai chi and meditation showed promising effects for hypertension and cholesterol [80], both risk factors for cardiovascular disease. Nutritional patterns also showed promising effects for the cardiovascular system, with the Mediterranean diet offering a protective effect on the incidence of heart failure, and DASH-type (dietary approaches to stop hypertension) diets showing potential in the primary prevention of heart failure [71]. In addition to behavioural factor recommendations, international organisations recommend the monitoring of metabolic risk factors through regular screening for hypertension (annual) [73,74]; diabetes and obesity [72,73]; and hypercholesterolemia, particularly in the context of vascular risk assessment, as well as estimating the individual 10-year risk factor of cardiovascular events [74]. Finally, specifically in men with a history of smoking, systematic screening for abdominal aortic aneurysm via ultrasound is recommended [73].

#### 3.6.3. Osteoarticular System

For the osteoarticular system, physical exercise was rated as effective in reducing the risk of osteoporosis and sarcopenia [80], with evidence further showing that these types of programs are superior for bone health in comparison to unimodal exercise [81]. Recommendations for addressing osteoporosis in postmenopausal women include screening with bone measurement, early management [73], the daily consumption of three dairy products (800 to 1200 mg), and supplementation in cases of sufficient intake of calcium or vitamin D (800 mg/day) [75].

#### 3.6.4. Genitourinary System

In terms of the genitourinary system, looking at nonpharmacological interventions for urinary incontinence, the WHO found improvements from prompted voiding (PV) and from pelvic floor muscle training (PFMT). PV led to decreased checks found to be wet and a reduction in daily incontinence episodes., PFMT led to decreased numbers of urinary incontinence episodes and symptoms, as well as an increase in patients’ perception of improvements in their incontinence and quality of life. The recommendation put forward was that “*PFMT, alone or combined with bladder control strategies and self-monitoring, should be recommended for older women with urinary incontinence…*” [76] (p. 24), with PV being recommended for people with cognitive impairment. Furthermore, international recommendations include the screening for cervical cancer until the age of 65 and prostate cancer screening until the age of 70 [72]

#### 3.6.5. Respiratory, Digestive, Renal, Lymphatic, and Immune Systems

Screening as a means for early detection and management is recommended for a range of cancers including lung cancer among ex-long-term smokers [73], breast cancer with biennial mammography until the age of 75, and colorectal cancer via a faecal occult blood test every two years, or via colonoscopy every 10 years [72]. Finally, yearly flu vaccinations, pneumococcal vaccination (with boosters every 10 years), and vaccination against herpes zoster are recommended between the ages of 65 and 79 [72]. Complete vaccination against SARS-CoV-2 (COVID-19) is likely to be recommended for several years.

### 3.7. Interventions for Caregiver Support

A caregiver is “a person in the immediate circle of an individual who is dependent on assistance for certain activities of daily living, who, on a non-professional and informal basis, provides him or her regular assistance, care or presence, of varying nature and intensity, intended to compensate for his or her…difficulties or to ensure his safety, the maintenance of his identity and social ties” [87] (p. 2, free translation from French). Much of the research on caregivers is focused on caregivers of people with dementia, and there is a lack of research comparing interventions, limiting the insights into their effectiveness [77]. Three focal points concerned interventions for caregiver support: respite care, psychological therapies and support, and training and education (Table 9).

Available research on respite care has sometimes provided conflicting findings [77,78]. For example, significant reductions were noted in caregiver depression and anger, with research also indicating, however, that caregiver quality of life was worse after respite use [77]. Day care services showed promising effects in reducing the burden of caregivers of people with dementia, but they also appeared to accelerate placement in nursing homes [78]. No significant effects of respite care on caregiver burden, anxiety, depression at short and long-term follow-ups were noted [77]. The lack and uncertainty of evidence led the WHO guideline development group to abstain from making a recommendation on respite care and the need for further research on this type of intervention, including the differentiation between different formats of respite care being highlighted [78].

Psychotherapy, specifically CBT and psychoeducation, were found to be beneficial for caregiver burden, depression, ability, and knowledge, with CBT also being effective for overall caregiver well-being [77]. Support and discussion groups led by professionals or peers were shown to be effective for caregiver burden and ability/knowledge, whereas training was effective for their subjective well-being [77]. As support and training interventions were shown to be beneficial for specific outcomes, it was suggested that they may be best used as part of multicomponent interventions. The recommendation put forward by the WHO was that “*psychological intervention, training and support should be offered to family members and other informal caregivers of care-dependent older people, particularly but not exclusively when the need for care is complex and extensive and/or there is significant caregiver strain*” [77] (p. 30). It was additionally recommended that new technologies accompany these interventions, in particular for online support groups and for training, the digital format of which may be particularly adapted to the situation of caregivers.

## 4. Discussion

The ageing world population combined with the associated risk of increased care dependency and the desire to age in place highlight the need for effective interventions that may facilitate ageing in a healthy manner. Using the latest framework put forward by the WHO, the present study provided a synthesis of effective interventions and international recommendations aimed at older persons for the maintenance or prevention of a further decline in domains which have been identified as central to healthy ageing. Specifically, it focused on intrinsic capacities, functional ability (ADL and IADL performance and social relationships), physiological system health, and caregiver support. In total, 38 documents were included reporting on over 400 individual studies. Fifty-four interventions were identified as effective or promising in their contribution to healthy ageing within particular domains. These were classified into nine broad categories according to their nature: physical activity, sociocultural, psychological, nutritional, home-based, preventive care, educational, cognitive, and other.

Certain types of interventions mapped relatively clearly onto specific health domains in terms of their benefit. For example, preventive care interventions, including screening and vaccinations, were related to physiological system health and to sensory capacities, whereas cognitive interventions were beneficial for cognitive capacity and ADL/IADL performance. Other types of interventions, however, were found to repeat across a range of domains, emphasising the inter-relatedness of the health domains [31]. Intergenerational interventions, for instance, showed benefits for cognitive, psychological, and locomotor capacities, as well as for the reduction in loneliness and the development of social relationships. Physical activity interventions were by far the most consistently impactful, showing strong positive effects on locomotor, cognitive, and psychological capacities, ADL/IADL performance, social relationships, and physiological systems. Of these, multimodal exercise was highlighted as effective for all domains it had an impact on and for a varied range of specific functions within each domain. This finding can be explained in terms of the nature of multimodal exercise, which targets different aspects of physical function, and it links in with previous research noting that multimodal exercise was the most frequently reported intervention in studies exploring long-term care interventions for older people [12]. Multimodal exercise can refer to a range of combinations of exercises. To maximise the benefits of such exercise and to facilitate its implementation, therefore, it would be interesting for research to explore and pinpoint the specific combination of exercises, whether one or more combinations, that brings about the most benefits [49].

When considering the impact of interventions based on the type of population in terms of their resource integrity or loss, certain noteworthy patterns emerge. For individuals not experiencing a loss of resources in their intrinsic capacities, the findings showed that a range of interventions can be beneficial in the maintenance of these capacities, notably physical activities, such as yoga, aquatic exercise, and tai chi, and sociocultural activities, including intergenerational programs, music, and art. The types of activities that can benefit this population, therefore, are broad, tapping on different domains. For those experiencing some resource loss, however, the findings suggest that the most beneficial interventions were ones which were more of a complex nature in the sense that they were multicomponent, often multidisciplinary, and tailor-made according to each individual’s needs. In the case of ADL and IADL performance, interventions including occupational therapy, whether in the form of home modifications, multidisciplinary rehabilitation, or preventive home interventions, were noted as those offering the greatest benefits. This highlights the importance of considering individual needs, goals, and environments within interventions. It also emphasises the benefits of a multidisciplinary and multicomponent nature in terms of physical, mental, and social well-being [88], particularly in the context of ageing in place. It is in line with person-centred approaches such as ICOPE, and relates back to the idea of integrated care in all settings of residence and across different societal levels (micro-, meso-, and macro-) for an effective implementation [89,90].

A lack of research comparing the effects of different interventions was noted for caregiver support, with often-conflicting findings being reported regarding the benefits of respite care [77,78]. At the same time, a consensus study of WHO global experts which identified the areas on which long-term care interventions for healthy ageing should focus, found that almost half of the interventions identified related to unpaid and paid caregivers’ support and training [91]. This places emphasis on the crucial role caregivers play in individuals’ ageing process, with unpaid caregivers (e.g., family members, relatives, and volunteers) being referred to as “the grassroots of care assistance” [91] (p. 300). The interventions agreed upon as being of high priority relate to training for the prevention or management of conditions which link back to intrinsic capacity and other domains discussed within the present review, such as fall prevention and response and the promotion of physical activity. Moreover, and given the well-documented negative impact of caregiving on overall health [23], social support was also noted as a priority long-term care intervention and one which was considered as efficient in the present paper [77]. Concerning respite interventions, the mixed findings reported in the literature could be discussed in terms of the point at which such assistance is sought. It is conceivable that caregivers, keen to provide all the support they can, seek out respite care at critical points when their own fatigue and general well-being have reached, or even surpassed, their limits. This could be linked to their commitment to the care receiver, to perceived moral obligations to provide support, or to a lack of knowledge on the available solutions and/or legal rights of care providers. A study carried out in Switzerland, for example, noted that despite the availability of respite care solutions, caregivers do not systematically seek them out, and that the need for such solutions came in third place after the need for information and for the recognition of their role as caregivers [92]. Therefore, although respite care solutions are critically essential, their uptake may increase from improving the accessibility of information on the offers available.

Taken together, the findings of the present work can be of use to the general public and to individuals working in a range of fields. For the older person and his/her family, having an understanding of which activities are likely to contribute to the health domain of interest may facilitate the choice of activity to engage in. For health and social care professionals, the findings can inform their daily practice and the recommendations they make to their clients/patients. Finally, policy makers and community representatives may strive to offer the types of activities highlighted in this review, aim to develop institutions dedicated to healthy ageing policies with permanent information desks, and/or engage in targeted health promotion and prevention campaigns. Considering the implementation of these findings in a worldwide context requires the consideration of several factors and the use of different strategies. First, the sociocultural and economic environment, including societal attitudes towards older people and local policies, plays a pivotal role in how such findings may best be implemented. For example, in certain contexts, work on ageism [93] may be a necessary first step before a more broad range of opportunities for maintaining an active lifestyle among older persons are put in place. Second, individual characteristics, such as health literacy, may involve the adaptation and delivery of such information through comprehensive language use, for example. Third, to achieve integrated care, the identification of barriers and enablers in interdisciplinary collaboration is an important step in ensuring the smooth operation of such a system [94]. In a practical sense, for example, the training of professionals in taking a health promotion/prevention stance, rather than a more curative, solution-based approach, may be necessary. Research on initiatives which have put the ICOPE framework in practice has now started emerging [95], allowing for recommendations on specific actions considered necessary for the implementation of this framework across different settings [89,96]. Such insights into the application of the ICOPE framework will help guide new initiatives and programs into a more effective operationalisation of this framework.

The present study provided a holistic overview of interventions and international recommendations related to healthy ageing, with the indication of their effectiveness offering important information which can directly be used and applied by the relevant stakeholders. The inclusion of solely high-level scientific evidence allowed for the synthesis of the most important research conclusions drawn on the topic in the last few decades, adding weight to the conclusions of this work. However, it also entails a limitation in that interventions without a robust research background were likely to have been omitted. For example, the use of animals, whether as part of formal therapy or as simple company [97,98], or cultural engagement [99,100,101,102], such as visiting museums, has been linked to benefits in a number of intrinsic capacities. Moreover, the choice to be pragmatic in the identification of included research meant that there was a lack of systematic searching of databases, giving rise to the possibility that other relevant studies were not captured.

## 5. Conclusions

As evidenced in this paper, there are many different types of activities older adults can choose to engage in which are likely to aid ageing in a healthy and balanced manner. This is an opportunity to be taken advantage of, both by the ageing individual and by communities. For the ageing individual, the ability to choose activities based on his/her interests and needs is positive for self-growth, motivation, and adherence. Communities play a crucial role here and can facilitate the endeavour to age healthily by offering a wide range of activities which are accessible: physically, in terms of proximity; financially; and with regard to the diffusion of information. Future research could test scientific findings and evidence-based recommendations through coherent and structured clinical itineraries, which would also allow for insight into the efficiency of interventions in real-life contexts. The challenges here are threefold. First, the provision of information must be accessible and comprehensible to all relevant stakeholders (i.e., ageing individuals and their families, healthcare professionals, and social workers). Second, existing resources must be identified and catalogued so that stakeholders may be guided to the scientific recommendations. Third, proximal multidisciplinary itineraries that are based on scientific evidence must be created to follow the ideology of person-centred care. This is precisely the aim of the next phase of the VIeSA project recently put in place in Geneva, Switzerland, which will integrate the findings of the present paper into a reference guide for use by professionals and individuals. This guide will then be used as a tool in the creation and implementation of personalized and inter-professional health itineraries [103,104].

## Figures and Tables

**Table 1:** Identified interventions for sensory capacities (vision and audition).

Intervention Type	Specific Intervention	ER	Specific Outcomes with Benefit
Preventive Care	Screening vs. no screening (vision and audition) [47,48]	NR	-
	Screening and provision of care [45]	***	Visual function and depression
	Screening and provision of hearing aids [46]	***	Social function, depression, and hearing aid use

Key: ER = effectiveness rating; NR = no recommendation; *** effective intervention.

**Table 2:** Identified interventions for locomotor capacity.

Intervention Type	Specific Intervention [Ref]	ER	Specific Outcomes with Benefit
Physical Activity	Physical activity interventions combined [80]	***	Improved physical performance, muscle function, muscle mass, gait, balance, agility, and flexibility
	Multimodal exercise ^ [49,50,81]	***	Balance, muscle strength, physical functioning, and functional lower extremity strength. Rate of falls, fall-related injuries, the fear of falling ^
	Aquatic exercise [79,80]	***	Balance, flexibility, muscular strength and mass, walking capacity, and fear of falling
	Yoga [80]	**	Balance and physical mobility
	Tai chi °^ [50,80]	**	Muscular strength, joint stiffness, physical mobility, and a reduction in the risk of falling °
		***	Balance ^
	Digital exercise [80]	**	Balance, physical performance, and physical activity uptake
	Supervised vs. unsupervised training programs [51]	***	Balance and muscle strength
	Sedentary behaviour reduction [80,81]	**	Physical function, activity level, risk of disability, muscle loss, and cardiometabolic capacities
	Progressive resistance training ^ [49,81]	***	Muscle strength and mass and body weight ^
	Dance [80]	**	A decrease in the fear of falling
Home-based	Multifactorial/unimodal multidisciplinary including OT ^ [50,64,80]	***	Gait, balance abilities, fear of falling, and rate of falls ^
	Home modifications ^ [50]	***	A decrease in the risk of falling ^
Sociocultural	Intergenerational activities [80]	***	Physical strength and a reduction in sedentary behaviour
	Music and singing [80]	**	A reduction in falls
Nutritional	Nutritional supplements ^ [52]	***	Muscle mass, strength, and physical performance ^
	Nutritional interventions with physical exercise °^ [52]	***	Muscle mass, strength, and physical performance ^ Sarcopenia prevention for healthy adults °
Other	The removal of psychotropic medications ^ [50]	***	A reduction in the rate of falls ^

Key: °^ intervention with benefits for people without and with limitations; ° intervention for people without limitations/not at risk of falling; ^ interventions with benefits for people with locomotor limitations or at high risk of falling; OT = occupational therapy; ER = effectiveness rating; *** effective intervention; ** promising intervention.

**Table 3:** Identified interventions for cognitive capacity.

Intervention Type	Specific Intervention	ER	Specific Outcomes with Benefit
Physical Activity	Physical activity interventions combined (including PRT and yoga) °^ [80]	***	Improvement in cognitive function and risk reduction in cognitive decline °^
	Aquatic exercise [79]	***	Improvements in visuospatial abilities, verbal short-term memory, and working memory
	Dance [55]	**	Global cognition and memory
Sociocultural	Intergenerational [80]	***	Global cognition
	Music (instrument playing) °^ [54]	***	Improvements in processing speed, global cognition, attentional control, and executive function °^
Cognitive	Cognitive stimulation ^ [53]	***	Global cognition ^
	Cognitive training °^ [53,80]	°*** ^**	Benefits in specific skills targeted °^
Psychological	Mindfulness [80]	**	Improvements in attention, memory, cognitive flexibility, verbal fluency, and executive function
	Meditation [80]	**	

Key: PRT = progressive resistance training; °^ intervention with benefits for people without and with limitations; ° interventions with benefits for people without limitations; ^ interventions with benefits for people with limitations; ER = effectiveness rating; *** effective intervention; ** promising intervention

**Table 4:** Identified interventions for vitality (nutrition).

Intervention Type	Specific Intervention [Ref]		Specific Outcomes with Benefit
Nutritional	Meal service [60]	***	Increase fruit consumption
	Food aid (free meals and education) [80]	**	Improved dietary intake
	Oral nutritional supplementation [57]	***	Weight gain and improved handgrip strength
Educational	Dietary education [58,59,60]	***	An increase in the consumption of fruit, vegetable, fibre, micronutrients, and protein

Key: *** effective intervention; ** promising intervention. Note: all interventions among people who were vulnerable, at risk of undernourishment, or undernourished.

**Table 5:** Identified interventions for psychological capacity.

Intervention Type	Specific Intervention [Ref]	ER	Specific Outcomes with Benefit
Physical activity	Physical activity interventions combined [80]	***	Reduction in anxiodepressive symptoms
	Aquatic exercise [79]	***	Anxiety, tension, and fatigue
	Tai chi [80]	**	Reduction in anxiodepressive symptoms
Sociocultural	Intergenerational programs [80]	***	Reduction in depressive symptoms
	Music and singing [80,82]	**	Reduction in anxiodepressive symptoms
	Music therapy ^ [63]	**	Reduction in depressive symptoms ^
Educational	Continued education and learning [82]	**	Increase in positive affect
Psychological	Mindfulness programs °^ [62,80]	**	Reduction in depressive symptoms and increased positive affect ° Depressive symptoms ^
	Behavioural activation ^ [61]		Reduction in the incidence of depressive symptoms ^
	Cognitive behavioural therapy ^ [61]		
	Life-review therapy ^ [61]	***	
	Problem-solving therapy ^ [61]		
	Interpersonal counselling ^ [61]		

Key: ER = effectiveness rating; ° interventions with benefits for people without limitations; ^ interventions with benefits for people with clinical depression or subthreshold depression/anxiety; °^ intervention with benefits for people with or without depression; *** effective intervention; ** promising intervention.

**Table 6:** Identified interventions for Activities of Daily Living and Instrumental Activities of Daily Living.

Intervention Type	Specific Intervention [Ref]	ER	Specific Outcomes with Benefit
Physical Activity	Accompanied by an occupational therapist [66]	***	ADL performance in frail older adults (moderate benefits)
	Multimodal exercise [49]	***	ADL performance
Home-based	Home modifications [66]	***	ADL performance in adults with difficulties in ADL (high benefits)
	Preventive home-based interventions including an occupational therapist [65]	***	IADL performance and functional disability
	Self-management home-based interventions including an occupational therapist [65]	***	IADL performance and social participation
	Home-based multidisciplinary rehabilitation [65]	***	Decelerated decline in IADL performance and improved mobility
	Multidisciplinary interventions including occupational therapy [64]	***	ADL performance
Cognitive	Functional cognitive interventions [65]	***	Cognition, with lasting effects on IADL performance

Key: ER = effectiveness rating; *** effective intervention; ADL = Activities of Daily Living; IADL = Instrumental Activities of Daily Living.

**Table 7:** Identified interventions for social relationships.

Intervention Type	Specific Intervention [Ref]	ER	Specific Outcomes with Benefit
Physical activity	Physical activity interventions combined [80]	***	Improvements in social health, socialization, and social connections
Sociocultural	Intergenerational programs [80,82]	***	Reduction in loneliness and the development/maintenance of social links. The development of meaningful roles
	Music and singing [67,80,82]	**	
	Arts and crafts [67,68]	**	
	Support groups/online [67]	**	
	Befriending programs [70,82]	**	
	Volunteering [70,82]	**	
Educational	Technology training/use in interventions [67,68,69,70,82]	***	Enables social participation and reduces isolation
	Social network/friendship training [67,70,82]	***	Development/maintenance of social links
Psychological	Cognitive behavioural therapy [70]	**	Loneliness reduction
	Mindfulness programs [70,80]	**	Loneliness reduction
Home-based	Multidisciplinary interventions including occupational therapy [64,65]	***	Increase in social participation

Key: ER = effectiveness rating; *** effective intervention; ** promising intervention.

**Table 8:** Identified interventions for physiological systems.

Intervention Type	Specific Intervention [Ref]	ER	Specific Outcomes with Benefit
Physical activity	Multimodal exercise [80,81]	***	Cardiovascular system aerobic activity (Vo2 max)
	Physical activity interventions combined [80]	***	Osteoarticular system (osteoporosis and sarcopenia)
	Tai chi [80]	**	Cardiovascular system (hypertension)
	Dance [80]	**	Cardiovascular system aerobic activity (Vo2 max)
	Sedentary behaviour reduction [80]	**	Cardiometabolic capacities
Preventive care	Screening cancers [72,73]	***	The early detection and treatment of lung, colorectal, breast, cervical, and prostate cancers
	Screening of risk factors [72,73,74]	***	The early detection of cardiovascular and metabolic risk factors (hypertension and obesity)
	Vaccinations [72]	***	Immunization against flu, pneumococcus, herpes zoster, and SARS-CoV-2 (COVID-19)
Nutritional	Mediterranean diet [71,74]	***	Cardiovascular-system-protective effect on the incidence of heart failure
	DASH diet [71]	**	Cardiovascular system primary prevention of heart failure
	Limiting alcohol consumption [72,74]	***	Cardiovascular health
Other	Smoking cessation (motivational and/or pharmacological) [74,80]	***	Cardiovascular, respiratory, and sensory health
	Prompted voiding [76]	**	Urinary incontinence episodes
	Pelvic floor muscle training [76]	***	Urinary incontinence episodes, symptoms, and quality of life

Key: ER = effectiveness rating; *** effective intervention; ** promising intervention.

**Table 9:** Identified interventions for caregiver support.

Intervention Type	Specific Intervention [Ref]	ER	Specific Outcomes with Benefit
	Support groups/online [77]	**	A decrease in burden and an increase in ability/knowledge
Educational	Caregiver training [77]	***	Increased subjective well-being
Psychological	Cognitive behavioural therapy [77]	***	A decrease in burden and depression and an increase in ability, knowledge, well-being
	Psychoeducation [77]	***	A decrease in burden and depression and an increase in ability and knowledge
Other	Respite care [77,78]	*	A reduction in depression, anger, burden, and quality of life. An acceleration of care receiver placement in nursing homes

Key: ER = effectiveness rating; *** effective intervention; ** promising intervention; * intervention with mixed findings/insufficient evidence for recommendation or determination of effectiveness.

## Data Availability

Not applicable.

## Supplementary Materials

The following supporting information can be downloaded at: https://www.mdpi.com/article/10.3390/ijerph20054382/s1, Table S1: Sensory capacities (vision & audition): main results and recommendations; Table S2: Locomotor capacity and the risk of falls: main results and recommendations; Table S3: Cognitive capacity: main results and recommendations: Table S4: Vitality: main results and recommendations: Table S5: Psychological capacity: main results and recommendations; Table S6: Activities of Daily Living and Instrumental Activities of Daily Living: main results and recommendations; Table S7: Social relationships: main results and recommendations; Table S8: Caregiver support: main results and recommendations; Table S9: Physiological system health: main results and recommendations. References included in the supplementary material are: [45,46,47,48,49,50,51,52,53,54,55,56,57,58,59,60,61,62,63,64,65,66,67,68,69,71,76,77,78,79,80,82,84].
