# Supplementary material for: Intrinsic Capacities, Functional Ability, Physiological Systems, and Caregiver Support: A Targeted Synthesis of Effective Interventions and International Recommendations for Older Adults
Source: Int J Environ Res Public Health. 2023 Mar 1;20(5):4382. doi: 10.3390/ijerph20054382 (PMC10002353; doi:10.3390/ijerph20054382)
Supplement: Supplementary file 1 [file ijerph-20-04382-s001.zip › ijerph-2155611-supplementary.pdf]

Table S1.
Sensory capacities (vision & audition): main results and recommendations

Ref	Topic [study design]<br>*Studies included*	Sample	General information<br>• Main findings<br>➔ Conclusions
[45]	Evidence profile: visual impairment [MA-GRADE]<br><br>*7 studies*<br>*(SR = 1; RCTs = 6)*<br><br>critical outcomes: visual acuity, vision-related QoL, self-reported improvement; important outcomes: social function, depression	*n* = 4037;<br>aged ≥ 60<br>visually impaired; uncorrected refractive errors	• Community case-finding: no evidence for any outcomes that vision screening (visual acuity testing/usual care/no vision or delayed screening) is effective<br><br>• Screening & immediate intervention for refractive error: improvement in visual function (SMD = 1.03; 95% CI: .42 to 1.65); depression (MD = -.74; 95% CI: -1.23 to -.26)<br><br>• Expedited vs routine surgery for cataract: improvement in visual acuity<br><br>➔ **WHO Recommendation: "**Older people should receive routine screening for visual impairment in the primary care setting, and timely provision of comprehensive eye care"
[47]	Screening for visual acuity in older adults [MA]<br><br>*25 studies (13 trials, 11 diagnostic accuracy studies, 1 SR)*	*n* = 33586<br>aged ≥ 65	• Screening in asymptomatic adults: no beneficial effects of screening for visual acuity, likelihood of vision disorders, quality of life<br><br>➔ **USPSTF Recommendation: "**the current evidence is insufficient to assess the balance of benefits and harms of screening for impaired visual acuity in older adults."
[46]	Evidence profile: hearing loss [MA-GRADE]<br><br>*4 studies*<br>*(SR = 1; RCTs = 3)*<br><br>critical outcomes: improvement in communication, social function, hearing use; important outcomes: depression QoL,	*n* = 6690;<br>aged ≥ 60<br>older adults with hearing loss	• Screening for hearing loss vs no screening: increased use of hearing aids RR = 2.8 (relative 95% CI: 1.41 to 3.68)<br><br>• Provision of hearing aid vs no hearing aid: improved social function MD = 33.4 (95% CI: 27.2 to 39.6); decreased depression MD = .08 (95% CI: .09 to 1.5); improved communication MD = 24 (95% CI: 17 to 31)<br><br>• Self-management support interventions vs control: reduced self-reported hearing handicap MD = -12.8 (95% CI: -23.11 to -2.48); increased use of verbal communication MD = .72 (95% CI: .21 to 1.23)<br><br>➔ **WHO Recommendation: "**Screening followed by provision of hearing aids should be offered to older people for timely identification and management of hearing loss"
[48]	Screening for hearing loss in older adults [MA]<br><br>*41 studies*	*n* = 26386<br>aged ≥ 50	• No differences in hearing function between screening and no screening at 1 yr<br>• Improvements in function from use of hearing aids limited to veteran samples<br><br>➔ **USPSTF Recommendation: "**the current evidence is insufficient to assess the balance of benefits and harms of screening for hearing loss in older adults"

Key: Ref = reference; [MA] systematic review and meta-analysis; [GRADE] Grading of Recommendations Assessment, Development, and Evaluation; RCT = randomized controlled trial; M = mean; MD = mean difference; SMD = standardized mean difference; CI = confidence interval; QoL = quality of life

Table S2.
Locomotor capacity and the risk of falls: main results and recommendations

Ref	Topic [study design]<br>*Studies included*	Sample	General information<br>• Main findings<br>➔ Conclusions
[49]	Evidence profile: mobility loss [MA-GRADE]<br><br>*8 SRs (older people without dementia)*<br><br><u>Critical outcomes</u>: muscle strength, balance, chair stand, timed up and go, physical functioning, ADLs, cognition; <u>important outcomes</u>: gait speed	*n* = 4022;<br>age range M = 70-81 history/risk of falls; physically frail; mobility/functional limitations; weak leg muscle strength; geriatric syndromes; balance limitations	In people with limitations in ADL performance:<br>• <u>Progressive resistance training vs control</u>: improved muscle strength (SMD = .32, 95% CI: .07 to .58) and chair stand performance (MD = -4.01 seconds, 95% CI: -7.3 to -.72)<br><br>• <u>Multimodal exercise vs control</u>: improved balance (SMD = .43, 95% CI: .15 to .7), muscle strength (SMD = .24, 95% CI: .02 to .45), and physical functioning (SMD = .25, 95% CI: .08 to .43); improved performance in chair stand (SMD = .41, 95% CI: .77 to .04), and ADLs (SMD = .73, 95% CI: .15 to 1.31)<br><br>• <u>Tai chi vs control</u>: improved balance (SMD = .25, 95% CI: .06 to .44)<br><br>➔ **WHO Recommendation:** "Multimodal exercise, including progressive strength resistance training and other exercise components (balance, flexibility, aerobic training) should be recommended for older people with declining physical capacity, measured by gait speed, grip strength and other physical performance measures"
[50]	Evidence profile: risk of falls [MA-GRADE]<br><br>*1 SR*	*n* = 79193; age ≥ 60 years (or mean age minus one SD > 60 years)<br>history of falls; risk factors for falls (e.g. vitamin D insufficiency/deficiency); hip or fall-related fracture; severe visual impairment; carotid sinus hypersensitivity; osteoporosis; osteopenia; Alzheimer's disease; chronic foot pain	• <u>Psychotropic medication withdrawal vs control</u>: decreased rate of falls (RR = .62, 95% CI: .16 to .73)<br>• <u>Home safety interventions vs control</u>: decreased rate of falls (RR = .62, 95% CI: .5 to .77) among people at higher risk of falls<br>• <u>Occupational therapy vs other professionals</u>: decreased risk of falls (RR = .69, 95% CI: .55 to .86)<br>• <u>Multifactorial interventions</u>: superior to control/usual care (RR = .76, 95% CI: .67 to .86), among people at higher risk of falls (RR = .77, 95% CI .66 to .90), containing assessment & active intervention (RR = .74, 95% CI: .61 to .89), or assessment and referral/ provision of information (RR = .82, 95% CI: .71 to .95)<br>• <u>Multimodal group or individual exercise</u>: overall decreased rate of falls (RR = .71, 95% CI: .63 to .82), also when delivered at home RR = .68, 95% CI: .58 to .80)<br>• <u>Tai chi</u>: effective in reducing risk of falls among people not at high risk of falling (RR = .59, 95% CI: .45 to .76)<br><br>**<u>WHO Recommendations</u>:**<br><br>➔ "Multifactorial interventions integrating assessment with individually tailored interventions can be recommended to reduce the risk and incidence of falls among older people"<br><br><u>For people at risk of falls</u>:<br>➔ "Medication review & withdrawal (of unnecessary/harmful medication) can be recommended"<br>➔ "Multimodal exercise (balance, strength, flexibility, functional training) should be recommended for … people at risk of falls"<br>➔ "Following a specialist's assessment, home modifications to remove environmental hazards that could cause falls"
[51]	Supervised vs unsupervised physical activity training programs [MA]<br><br>*11 RCTs*	*n* = 621;<br>age range = 65-81 healthy individuals (absence of cognitive limitations/illness), some with limited performance on specific tests (e.g. leg extensor torque)	• <u>Supervised balance/resistance training superior to unsupervised</u>: static steady-state balance (M SMD_{bs} = .28[*]); dynamic steady-state (M SMD_{bs} = .35[*]); proactive balance (M SMD_{bs} = .24[*]); muscle strength/power (M SMD_{bs} = .51[*])<br>• <u>Dose-response relationships</u>: 10-29 additional supervised sessions = largest benefits for all measures Small amounts of supervision within unsupervised programs can show added benefits<br><br>**Conclusions:**<br>➔ Supervised training has greater benefits for balance and muscle strength than unsupervised<br>➔ Recommended to include supervised sessions (2 of 3 sessions/week)

[80]*	Effective interventions for healthy aging [SR]<br><br>*31 studies (SRs = 28; systematic reports by French public health organizations = 3)*<br><br>Outcomes: physical, mental, social health	*n* = NR<br>aged ≥ 55 years<br>independent community-dwelling	• Interventions for healthy individuals: physical activity programs; mind-body; cognitive stimulation; intergenerational; ICT; cultural<br>• Interventions for individuals at health/social risk: home visits; mutual aid; nutritional assistance<br>• Interventions for healthy & at risk individuals: counselling<br>➔ **Conclusions:** Effective and promising interventions act on several health determinants, justifying a global approach for health promotion and prevention among older people
[52]	Nutritional interventions to prevent functional decline in community-dwelling older adults [SR]<br><br>*28 RCTs*	*n* = 3631;<br>aged ≥ 65 years healthy; frail; sarcopenic; low serum vitamin D levels; obese; undernourished	• Unimodal nutritional interventions: some benefits in muscle mass, muscle strength & physical performance among frail & sarcopenic individuals. Little benefits for healthy people.<br>• Nutritional & physical interventions: efficient in preventing sarcopenia among healthy individuals; efficient for muscle mass, muscle strength and physical performance in sarcopenic/frail individuals.<br>• Multimodal (> 2) interventions: improvements in handgrip strength & SPPB scores among frail individuals.<br>**Conclusions:**<br>➔ Nutritional supplementation efficient for muscle mass & strength, physical performance in frail individuals<br>➔ Additional benefits when adding physical exercise, especially in frail & sarcopenic individuals<br>➔ Multimodal interventions most efficient among frail individuals

Key: Ref = reference; [SR] systematic review; [MA] systematic review and meta-analysis; [GRADE] Grading of Recommendations Assessment, Development, and Evaluation; *article addressing a range of health domains; RCT = randomized controlled trial; CBT = Cognitive-Behavioral Therapy; SPPB = Short Physical Performance Battery; M = mean; SD = standard deviation; MD = mean difference; SMD = standardized mean difference; M SMD_{bs} = mean standardized mean difference between subjects; RR = rate ratio; CI = confidence interval

Table S3.
Cognitive capacity: main results and recommendations

Ref	Topic [study design]<br>*Studies included*	Sample	General information<br>• Main findings<br>➔ Conclusions
[53]	Evidence profile: cognitive impairment [MA-GRADE]<br><br>*2 SRs*	*n* = 2017;<br>aged ≥ 60<br>MCI; dementia	• Cognitive stimulation therapy vs control: improved cognitive function (MMSE SMD = .21, 95% CI: .03 to .39; ADAS-cog SMD = -.3, 95% CI: -.48 to -.13)<br>• Cognitive training and/or cognitive rehabilitation vs control: *ns*<br>• Cognitive training vs no treatment: improved memory (immediate recall) in people with MCI (SMD = .5, 95% CI = .02 to .98)<br><br>➔ **WHO Recommendation: "**Cognitive stimulation can be offered to older people with cognitive impairment, with or without diagnosis of dementia"
[54]	Instrument playing as cognitive intervention [MA]<br><br>*8/10 studies~ (RCTs = 6; controlled clinical trial = 1; study with control group =1)*	*n* = 635<br>aged ≥ 60<br>healthy, individuals with MCI	Five types of interventions: instrumental improvisation, piano instruction, percussion while memorizing rhythms/reading music scores, group instrument playing in specified rhythm/timing, instrument playing while walking.<br><br>Three levels of cognitive engagement: immediate with addition of cognitive tasks; immediate with addition of motor tasks; sustained (e.g. memorization of music)<br><br>• Instrument playing: Small effect sizes for general cognition (*d* = .28), memory (*d* = .26), verbal fluency (*d* = .19), attentional control (*d* = .22), executive function (*d* = .25), visuospatial perception (*d* = .19). Large effect size for processing speed (*d* = .94)<br>• Immediate cognitive engagement: beneficial for attentional control (SMD = .32, 95% CI: .02 to .63), general cognition (SMD = .40, 95% CI: .16 to .64); small *ns* effect sizes for verbal fluency, visuospatial perception<br>• Sustained engagement: beneficial for executive function (SMD = .52, 95% CI: .12 to .92), memory (SMD = .94, 95% CI: .39 to 1.49), processing speed (SMD = .94, 95% CI: .52 to 1.35)<br><br>➔ **Conclusions:** Different types of instrument playing may target specific cognitive domains among adults with different levels of cognitive ageing
[55]	Dance interventions on global cognition [MA]<br><br>*13 studies (RCTs = 10; quasi-experiment = 3)*	*n* = 1605;<br>age range = 59-95<br>healthy; 2 studies in people with MCI; 1 study in people with metabolic syndrome	Different types of dance investigated, including ballroom, contemporary, tango, waltz, and folk dances<br><br>• Global cognition: SMD = 1.95** (MMSE MD = 1.57**; SCEF MD = 33.25***)<br>• Executive functioning: (SMD = .17, *ns*)<br>• Memory: significant benefits in dance groups (memory function; delayed recall; word list recognition; short-term auditory-verbal memory)<br><br>➔ **Conclusions:** Dance may be an effective and safe intervention for cognitive function improvements
[56]	Real-world interventions for cognitive ageing in healthy older adults [MA]<br><br>*43 RCTs*	*n* = 5060;<br>age > 60 years old<br>healthy older adults	Four types of interventions: physical (resistance training, yoga, dance, aerobic & aquatic exercise, Tai Chi); cognitive (video games, computer training, reading, arithmetic problem-solving); mixed (physical & cognitive); intergenerational (assisting schoolchildren, use of Facebook to maintain/enhance cognitive function)<br><br>• Physical activity interventions compared to all others: visuospatial abilities *M* = .25*; digit span forward *M* = .91*<br>• Cognitive activity interventions compared to all others: *ns*<br>➔ **Conclusions:** Physical activity interventions are most effective for cognition, and visuospatial abilities may be most susceptible to improvements with physical activity

Key: Ref = reference; [SR] systematic review; [MA] systematic review and meta-analysis; [GRADE] Grading of Recommendations Assessment, Development, and Evaluation; ; ~some studies excluded from current review due to pathology/settings: *n* adjusted to reflect actual number of participants in current review; RCT = randomized controlled trial; MCI = Mild Cognitive Impairment; MMSE = Mini-Mental State Examination; ADAS-cog = Alzheimer's Disease Assessment Scale cognitive subscale SCEF= Scale of Elderly Cognitive Function; M = mean; SD = standard deviation; MD = mean difference; SMD = standardized mean difference; CI = confidence interval; ***$p < .001$; **$p < .01$; *$p < .05$; *ns* = non-significant

Table S4.
Vitality: main results and recommendations

Ref	Topic [study design]<br>*Studies included*	Sample	General information<br>• Main findings<br>➔ Conclusions
[57]	Evidence profile: malnutrition [MA-GRADE]<br><br>*2 SRs; 1 MA*	*n* = NR<br>older people at risk of undernourishment or undernourished	• <u>Dietary advice alone</u> = *ns*; mealtime enhancement: weight gain in hospital settings for people undernourished/at risk<br><br>ONS with/without dietary advice<br>• <u>For undernourished people</u>: weight gain in community settings (MD = 3.17^{**}), hospitals/long-term care (MD = 1.8^{**}); reductions in mortality (RR = $.84^{*}$)<br><br>• <u>At risk for undernutrition in hospitals/long-term care</u>: weight gain (MD = $.26^{***}$); hand-grip strength (SMD = $.18^{*}$); no benefits for community-dwelling people at risk for undernutrition<br><br>➔ **WHO recommendation: "**Oral supplemental nutrition with dietary advice should be recommended for older people affected by undernutrition"
[58]	Educational interventions on nutrition [MA]<br><br>*9/11 RCTs~*	*n* = 3893<br>aged ≥ 50<br>community-dwelling; diabetes;; sedentary	Individual / group interventions with participants taking one/more of following materials home: educational book, goal setting exercises, written materials (e.g. recipes, physical activity handouts, lesson messages). Some studies included phone call follow-ups to improve adherence & give individualized recommendations<br><br>• Interventions efficient in increasing FV; fiber, micronutrients<br><br>• <u>Meta-analysis increases in intake of</u>: FV (SMD = .20, 95% CI: .12 to .28); V (SMD = .25, 95% CI: .15 to .34); F (SMD = .18, 95% CI: .08 to .27); FI (SMD = .27, 95% CI: .18 to .36)<br><br>➔ **Conclusions:** Nutritional interventions including educational element are efficient in increasing F, V, FI intake
[59]	Health-promotion theories in nutritional interventions [SR]<br><br>*8 RCTs*	*n* = 3662;<br>age range = 60-85<br>home-dwelling, healthy	• <u>Nutritional interventions</u>: overall successful in increasing F, FV & protein intake; nutritional knowledge & attitudes<br><br>**Conclusions:**<br>➔ Specific nutritional interventions are more effective than generic<br>➔ Multiple educational sessions more effective than single ones
[60]	Behavioral interventions promoting healthy eating [SR]<br><br>*13/16 studies (RCTs = 8; quasi-exp. = 4; pilot trial = 1)*	*n* = 21,258;<br>age range = 50-90<br>with risk factors for chronic disease; frail	Three types of interventions (methods): dietary education (health program; tailored newsletter; nutrition advice/counselling on benefits of healthy eating); meal service (healthy food service, repeated exposure to food); multicomponent (healthy meal provision & dietitian advice)<br><br>• <u>Dietary education</u>: 3/7 studies showed benefits: increased FV frequency & intake, nutrition status, stages of change<br><br>• <u>Meal service</u>: increased F consumption<br><br>• <u>Multicomponent</u>: main contribution to reduction of risk of chronic disease (diabetes, CVD)<br><br>➔ **Conclusions:** Dietary education & healthier meal services can lead to improved dietary quality

Key: Ref = reference; [SR] systematic review; [MA] systematic review and meta-analysis; [GRADE] Grading of Recommendations Assessment, Development, and Evaluation; ; ~some studies excluded from current review due to pathology/settings: *n* adjusted to reflect actual number of participants in current review ; RCT = randomized controlled trial; NR = not reported; MD = mean difference; $^{***}p < .001$; $^{**}p < .01$; $^{*}p < .05$; *ns* = non-significant; ONS= oral nutritional supplement; F = fruit; V = vegetable; FV= fruit/vegetable; FI = fiber;

Table S5.
Psychological capacity: main results and recommendations

Ref	Topic [study design]<br>*Studies included*	Sample	General information<br>• Main findings<br>➔ Conclusions
[61]	Evidence profile: depressive symptoms [MA-GRADE]<br><br>*3 SRs*	*n* = 9651<br>age ≥ 60 years old<br>clinical depression; threshold depression	• Behavioral activation vs usual care: depressive symptoms reduced in individuals with depression (SMD = -.81, 95% CI: -1.22 to -.40), also potentially beneficial in cases where antidepressants were ineffective<br><br>• Psychological interventions (CBT, problem-solving, behavioral activation) vs usual care: effective among people with subthreshold depression (SMD = -.3, 95% CI: -.46 to -.14), reduce incidence of depressive disorder at 2 months follow-up (RR = .5, 95% CI: .26 to .97), 6 months (RR = .74, 95% CI: .45 to 1.22), 12 months (RR = .88, 95% CI: .64 to 1.21)<br><br>➔ **WHO recommendation:** Older adults who are experiencing depressive symptoms can be offered brief, structured psychological interventions… delivered by health care professionals with a good understanding of mental health care for older adults.
[79]*	Effects of aquatic physical exercise on neuropsychological factors [SR]<br><br>*13/16 clinical trials~ (RCTs =13)*<br><br>Outcomes: behavior, cognition, psychological, mental health	*n* = 1637; age range = 65-88 (M = 71.3)<br>majority female; sedentary without cognitive impairment; history of falls; osteoarthritis	• Healthy & sedentary individuals: benefits on QoL, fear of falling, cognition, mood state (fatigue; tension), anxiety, health locus of control<br><br>**Conclusions:**<br>➔ Beneficial impact of aquatic exercise, regardless of protocol, with high-intensity exercises most impactful<br>➔ Beneficial effects mostly among healthy and sedentary people
[62]	Effects of mindfulness-based stress reduction (MBSR) on depression, anxiety, and stress [MA]<br><br>*6 RCTs*	*n* = 553;<br>age range = 62-82<br>healthy; depressive/anxiety disorder	• MBSR vs waiting list controls: large positive effects among clinically depressed older people<br><br>• Active control: superior to MBSR for depression<br><br>• No evidence for stress/anxiety reduction or long-term maintenance of positive effects<br><br>➔ **Conclusions:** MBSR beneficial in clinical samples not undergoing other treatment
[63]	Music therapy for depression [MA]<br><br>*12/19 RCTs~*	*n* = 1041;<br>age range = 60-92<br>depression (major/minor), risk of falls, osteoporosis	• Music therapy added to standard care: benefits for reduction of depressive symptoms<br><br>• Music therapy vs standard care = *ns*<br><br>➔ **Conclusions:** Music has, to some extent, a positive impact on depressive symptoms

Key: Ref = reference; [SR] systematic review; [MA] systematic review and meta-analysis; [GRADE] Grading of Recommendations Assessment, Development, and Evaluation; *article addressing a range of health domains; ~some studies excluded from current review due to pathology/settings: *n* adjusted to reflect actual number of participants in current review; RCT = randomized controlled trial; SMD = standardized mean difference; RR = relative risk; CI = confidence interval; *ns* = non-significant

Table S6.
Activities of Daily Living and Instrumental Activities of Daily Living: main results and recommendations

Ref	Topic [study design]<br>*Studies included*	Sample	General information<br>• Main findings<br>➔ Conclusions
[64]	Occupational therapy and functioning in frail older people [MA]<br><br>*12 RCTs*<br><br><u>Primary outcomes</u>: mobility, ADL functioning, social participation<br><u>Secondary</u>: fear of falling, cognition	*n* = 3163; age range = 60-95 community-dwelling people with disabilities, difficulties with ADLs, risk of falls, or both	Multidisciplinary teams: OT, physiotherapists, nurses, dietitians, social workers, GPs, geriatricians, case managers.<br><br>• <u>Primary outcomes</u>: ADLs: SMD = -.30**; social participation: SMD = -.44***; mobility: -.45**<br><br>• <u>Secondary outcomes</u>: showed positive trends, with fear of falling being significant<br><br>➔ **Conclusions:** Strong evidence that occupational therapy can contribute to better functioning of community-dwelling older adults
[65]	Occupational therapy interventions to improve IADLs [SR]<br><br>*14 studies*<br>*(RCTs = 13; one-group non-randomized = 1)*	*n* = 5675 aged ≥ 65 years community-dwelling, MCI included, but not dementia	Four types of interventions for improvement in IADLs:<br>• <u>Cognitive</u>: functional interventions superior to cognitive training; interventions focused on memory, reasoning, & processing speed lasting benefits on IADL performance (at 10 year follow-up)<br><br>• <u>Self-management</u>: significant improvements in IADLs, social participation<br><br>• <u>Prevention</u>: reduction in functional disability, greater IADL performance<br><br>• <u>Home-based multidisciplinary rehabilitation</u>: less decline in IADL performance, better engagement/ performance in domestic & outdoor activities, improved mobility<br><br>➔ **Conclusions:** Strong evidence for tailored interventions to enhance IADL performance
[66]	Occupational therapy interventions to improve ADLs [SR]<br><br>*43 studies*<br>*(RCTs =34; non-randomized with 2 or more groups = 4; one group non-randomized = 4)*<br><br><u>Outcomes</u>: performance of basic ADLs	*n* = 7595; M age range = 60s-80s no ADL difficulty, with ADL difficulty, receiving restorative home care services, frail, discharged to home from hospital, history of hip fracture	Six types of interventions: physical, home-based, multicomponent, additional OT, inpatient therapy, multidisciplinary team.<br><br>• <u>Physical exercise</u>: beneficial among frail older adults (moderate evidence); task-specific exercise beneficial for people with difficulties in ADL (moderate evidence)<br><br>• <u>Home-based</u>: high benefit for people with ADL difficulty<br><br>➔ **Conclusions:** Physical exercise interventions most beneficial for frail older people; home-based interventions for those with ADL difficulty.

Key: Ref = reference; ADL = Activities of Daily Living; IADL = Instrumental Activities of Daily Living; [SR] systematic review; [MA] systematic review and meta-analysis; ~some studies excluded from current review due to pathology/settings: *n* adjusted to reflect actual number of participants in current review; RCT = randomized controlled trial; SMD = standardized mean difference; RR = relative risk; CI = confidence interval; ; $^{***}p < .001$; $^{**}p < .01$; *ns* = non-significant

Table S7.
Social relationships: main results and recommendations

Ref	Topic [study design]<br>*Studies included*	Sample	General information<br>• Main findings<br>➔ Conclusions
[82]	Older people: independence & mental well-being [SR]<br><br>*86 studies*	*n* > 16600<br>age range = 50-100	• Majority of interventions to protect mental wellbeing, less on independence<br>• Interventions found to improve access to social contacts and networks, participation in social activities, locating individuals to refer them to activities and friendship programs.<br>• Volunteering and intergenerational activities reduce social isolation and challenge negative stereotypes/attitudes towards older people.<br>• Educational activities remotely/in person show potential to improve wellbeing and positive affect<br>• Some evidence found for singing (though unclear if this is due to the activity or social nature of it), creative arts, information and communication technologies.
[67]	Educational interventions for alleviating loneliness [SR]<br><br>*22/34 studies~ (RCTs = 11; RNC = 2; NRC = 4; NRNC = 4, matched controlled =1)*	*n* = 4478;<br>age range = 50-97<br>community-dwelling; healthy; cognitively intact; handicapped; widowed; single	Psychosocial element included in most interventions: social skills practice, social interaction facilitation, social network development<br><br>• Group interventions: 3/15 effective psychosocial: health promotion facilitating social networks support; shared activities: group exercise delivered by professional; visual art discussions; 9/15 promising (shared activities: chorale participation: professionally conducted, with public performances; foster grandparent: caring for disabled person, daily discussions with peers; educational: self-management of well-being; psychosocial: friendship enrichment; art, group exercise, therapeutic writing; group activities, discussions, lectures, exercise; phone support group; support group sessions)<br><br>• One-on-one interventions: 2/7 effective (psychosocial: teaching care receivers how to maximize relationship with caregivers; computer training: lessons on using email, internet by volunteers); 2/7 promising (psychosocial: phone crisis program with assessment, tailored service, supportive therapy for communication skills; sensory technological aids for hearing loss with "hearing diary")<br><br>➔ **Conclusions:** Loneliness can be reduced by educational interventions focused on maintenance & enhancement of social networks
[68]	Interventions targeting loneliness and social isolation [SR]<br><br>*19/20 studies~ (quantitative = 14/15; qualitative = 5)*	quantitative studies:<br>*n* = 1965;<br>age range = 53-114<br>community-dwelling adults: no particular risk; at risk for social isolation; lonely<br>qualitative studies:<br>*n* = 240; aged > 57	• Types of interventions: social support (discussion, counselling, therapy, education) within complex interventions; social activities, physical activities, technological<br><br>• At least one significant finding for loneliness/isolation: in 6/11 group, 1/4 mixed, 3/3 individual interventions<br><br>➔ **Conclusions:** New technologies and community engaged arts promising for reduction of social isolation & loneliness
[69]	Efficacy of interventions for social isolation [SR]	*n* = 3832;<br>age range M = 53-85	Three types of interventions (with subtypes): group (activities provided; training support; remote service), individual (face-to-face; remote service), mixed (remote

	22/24 RCTs~	no mental illness or cognitive impairment; disabled; chronic rheumatism	service; interview). Delivered by: health commissioners, social workers, teachers, students, experts<br>• <u>Group interventions & individual intervention interviews</u>: effective for structural social support<br>• <u>Group interventions (training support) & Mixed interventions</u>: effective for functional social support<br>• <u>Increased effectiveness</u>: interventions targeted on people with loneliness in social/ public locations; active participation of older people; delivery by professionals vs teachers/students<br>➔ **Conclusions:** Group & mixed interventions and use of modern technology for remote services helpful for decreasing social isolation

Key: Ref = reference; [SR] systematic review; [MA] systematic review and meta-analysis; ~some studies excluded from current review due to pathology/settings: *n* adjusted to reflect actual number of participants in current review; RCT = randomized controlled trial; SMD = standardized mean difference; RR = relative risk; CI = confidence interval; *ns* = non-significant

Table S8.
Caregiver support: main results and recommendations

Ref	Topic [study design]<br>*Studies included*	Sample	General information<br>• Main findings<br>➔ Conclusions
[77])	Evidence profile: caregiver support [MA-GRADE]<br><br>*3 SRs*<br><br><u>Critical outcomes</u>: caregiver burden, caregiver depression, care recipients' symptoms<br><u>Important</u>: well-being, ability/knowledge, QoL, anger, anxiety	*n* = > 7000<br>Family caregivers of people aged 60 and over	• <u>Psychotherapy (mainly CBT)</u>: Efficient for all outcomes: caregiver burden (SMD = -.22, 95% CI: -.41 to -.03), depression (SMD = -.27, 95% CI: -.45 to -.09), well-being (SMD = .52, 95% CI: .08 to .96), ability/knowledge (SMD = .38, 95% CI: .14 to .61)<br><br>• <u>Psychoeducation</u>: Beneficial for caregiver burden (SMD = -.12, 95% CI: -.24 to -.01), depression (SMD = -.23, 95% CI: -.38 to -.08), ability/ knowledge (SMD = -.23, 95% CI: .24 to .51)<br><br>• <u>Support and training interventions</u>: Support and discussion groups led by professionals/peers: beneficial for caregiver burden (SMD = -.35, 95% CI: -.6 to -.1), ability/knowledge (SMD = .53, 95% CI: .3 to .78). Training beneficial for caregiver subjective well-being (SMD = .74, 95% CI: .06 to 1.42)<br><br>• <u>Respite care</u>: reduction in caregiver depression (SMD = -0.32, 95% CI: -0.62 to -0.02) and anger (SMD = -.38, 95% CI: -.60 to -.17). No other benefits found, caregiver QoL worse after respite use. Lack of studies on respite care, including different types of respite care service (e.g. day care, residential home)<br><br>• Support and training interventions are beneficial for specific outcomes and may be best as part of multicomponent interventions<br>• Much of research focused on caregivers of people with dementia, lack of studies comparing interventions limiting insights into effectiveness<br><br>➔ **WHO recommendation:** "Psychological intervention, training and support should be offered to family members and other informal caregivers of care-dependent older people, particularly but not exclusively when the need for care is complex and extensive and/or there is significant caregiver strain"
[78]	Effectiveness of respite care in supporting informal caregivers of persons with dementia [SR]<br><br>*17 studies (RCTs = 3; experimental design = 6; longitudinal = 5; prospective = 3)*	*n* = 4000<br>caregivers of older people with dementia	• <u>Day care services</u>: decrease caregiver burden & behavioral issues in people with dementia. Also accelerate placement in nursing home<br><br>• <u>Temporary residential admission</u>: mixed results with negative effects on caregivers & care recipients<br><br>• <u>Evidence for respite care</u>: lacking, but promising effects for caregiver morbidity & mortality (1 study)<br><br>➔ **Conclusions:** Need further research for impact of respite care, including in-home, on caregivers & care recipients

Key: Ref = reference; [SR] systematic review; [MA] systematic review and meta-analysis; [GRADE] Grading of Recommendations Assessment, Development, and Evaluation; ; ~some studies excluded from current review due to pathology/settings: *n* adjusted to reflect actual number of participants in current review; RCT = randomized controlled trial; ~some studies excluded from current review due to pathology/settings: *n* adjusted to reflect actual number of participants in current review; QoL = quality of life; CBT = Cognitive-Behavioral Therapy; M = mean; SMD = standardized mean difference; CI = confidence interval

Table S9.
Physiological system health: main results and recommendations

Ref	Topic [type of study]<br>*Studies included*	Sample	General information<br>• Main findings<br>➔ Conclusions
[76]	Evidence profile: urinary incontinence [MA-GRADE]<br><br>*5 SRs*	*n* = 4104<br>older adults with urinary incontinence	• Prompted voiding vs no prompted voiding: decreased checks that were wet (MD = -12, 95% CI: -18.79 to -5.21), number of incontinence episodes (MD = -.92, 95% CI: -1.32 to -.53); self-initiated toileting (MD = -1.9, 95% CI: -2.29 to -1.51)<br><br>• Pelvic-floor muscle training (PFMT) vs control: decreased number of incontinence episodes (WMD = -3.63 (95% CI: -5.19 to -.99), increased patients' perception of improvement in incontinence (RR = 4.15, 95% CI = 2.70 to 6.37)<br><br>• PFMT vs no treatment: increased patient perceived cure (RR = 5.34, 95% CI: 2.78 to 10.26) & QoL (MD = -24.92, 95% CI: -36.06 to -10.78); decreased incontinence symptoms (MD = -34.16, 95% CI: -47.45 to -20.88)<br><br>➔ **WHO recommendation:** "Pelvic floor muscle training (PFMT), alone or combined with bladder control strategies and self-monitoring, should be recommended for older women with urinary incontinence (urge, stress or mixed)."
[84]	Guidelines on physical activity and sedentary behavior [SR]<br><br>*21 systematic reviews (111 RCTs)*	*n* = 9082<br>older adults aged > 64 years old	➔ **WHO recommendation:** "All older adults should undertake regular physical activity<br>➔ **WHO recommendation:** "Older adults should do at least 150–300 minutes of moderate-intensity aerobic physical activity; or at least 75–150 minutes of vigorous-intensity aerobic physical activity; or an equivalent combination of moderate- and vigorous intensity activity throughout the week for substantial health benefits.<br>➔ **WHO recommendation:** As part of their weekly physical activity, older adults should do varied multicomponent physical activity that emphasizes functional balance and strength training at moderate or greater intensity, on 3 or more days a week, to enhance functional capacity and to prevent falls.
[71]	Dietary patterns in primary prevention of heart failure [SR]<br><br>*12/14 studies (RCTs = 2; cohort studies = 7; cross-sectional = 2)*<br><br>Outcomes: primary prevention of heart failure	*n* = 187,846;<br>M age range = 50-71 patients without previous cardiovascular disease	Studies on Mediterranean, DASH, vegetarian, and Paleolithic diets<br><br>• Paleolithic diet: no significant effects<br>• Vegetarian diet: little research, but benefits on left ventricular diastolic function<br>• DASH diet: Conflicting results, but potential benefits in primary prevention of HF<br>• Mediterranean diet: protective effect on incidence of heart failure<br><br>**Conclusions:**<br><br>➔ Adoption of Mediterranean or DASH-type diets may be beneficial for the prevention of HF<br>➔ Results should be taken with caution due to low-quality evidence

Key: Ref = reference; [SR] systematic review; [GRADE] Grading of Recommendations Assessment, Development, and Evaluation; MD = mean difference; WMD = weighted mean difference; RR = relative risk; QoL = quality of lie; DASH = dietary approaches to stop hypertension; HF = heart failure
